# Reconstruction of the corneal epithelium with induced marrow mesenchymal stem cells in rats

**Published:** 2010-07-14

**Authors:** Ting-Shuai Jiang, Li Cai, Wei-Ying Ji, Yan-Nian Hui, Yu-Sheng Wang, Dan Hu, Jie Zhu

**Affiliations:** Department of Ophthalmology, Xijing Hospital, Fourth Military Medical University, Xi’an, Shannxi, China

## Abstract

**Purpose:**

To explore the feasibility of bone marrow mesenchymal stem cells (MSCs) transdifferentiating into corneal epithelial cells in a limbal stem cell deficiency (LSCD) model in rats.

**Methods:**

Rat MSCs were isolated and purified using a gradient isolation procedure. The cells were induced by rat corneal stromal cells (CSCs) in a transwell co-culture system. The induced MSCs were identified by immunofluorescence staining, flow cytometry, and scanning electron microscopy (SEM). A corneal LSCD model was produced in the right eyes of 48 rats by alkali injury. The eyes of 12 rats without any transplant served as controls (Group 1). Amniotic membranes (AM; Group 2), uninduced MSCs (Group 3), or MSCs induced by CSCs (Group 4), were transplanted onto the cornea of the model (n=12 each). The therapeutic effects of the four groups were evaluated by slit lamp observation, hematoxylin and eosin staining, immunohistochemistry staining, and confocal laser corneal microscopy.

**Results:**

Cultivated MSCs were positive for CD29, CD44, and CD90, but negative for CD34, CD45, CD133, and CK12, with typical MSCs characteristics revealed by SEM. After co-culture with CSCs, the induced MSCs expressed positive staining for CK12 with corneal epithelial cell characteristics confirmed by SEM; the induced MSCs were unchanged on the amnion. Compared with the other three groups, the corneal opacity, fluorescence staining, and neovascularization grades were significantly decreased in the induced MSCs group, both on postoperative week four and ten.

**Conclusion:**

MSCs induced by CSCs can transdifferentiate into corneal epithelial cells in vitro. The induced MSCs on an amniotic membrane have remarkable effects on the treatment of corneal alkali burn and the reconstruction of the corneal surface of rats.

## Introduction

Corneal damage can result from a variety of corneal disorders including keratitis, Stevens-Johnson syndrome, and chemical, mechanical, or thermal injuries. In a severe injury, both the eye limbus and central epithelium could be lost, accompanied by inflammation, neovascularization, and conjunctivalization [1,2]. The prognosis of corneal damage in many cases depends on the extent of deficiency in the limbal stem cells (LSCs). Studies show that LSCs are a major renewable source of corneal epithelium. Studies have further confirmed that LSCs have a greater growth potential in explant cultures and higher clonogenicity when co-cultured on 3T3 fibroblast feeder layers [3].

A lack of LSCs will lead to severe ocular surface abnormalities. In a severely injured cornea, both limbal and central epithelia are absent and conjunctival epithelial cells invade the corneal surface, resulting in an abnormal conjunctiva covering the corneal surface. This process is accompanied by chronic inflammation, persistent epithelial defects, stromal scarring, and neovascularization [4], producing decreased visual acuity and photophobia. Limbal grafts are still the prevailing option, although some potential disadvantages or complications may result from it. An autologous limbal graft is only considered as a potential alternative in cases of unilateral lesions, and limbal sampling bears the potential risk of causing LSC deficiency (LSCD) in the healthy contralateral eye [5]. A limbal allograft with systemic immunosuppression is another option. However, the risk of significant side effects from long-term immunosuppression is a major drawback of that technique. It has been reported that corneal limbal epithelial cells cultured ex vivo on a human amniotic membrane (AM) can repair an injured ocular surface [2,6,7]. A new therapeutic approach for patients having LSCD was described by Pellegrini and colleagues [8,9]. They reduced the surface area required for an autologous limbal biopsy by expanding these human cells in culture and seeding them over a fibrin gel before grafting.

However, because of the exiguity of autograft material, it is not suitable for binoculus sufferers who are unwell as it risks damaging the comparatively healthy eye. Therefore, the key method to resolving this problem is to look for cells that can be easily collected with sufficient quantity and that have the ability to replace LSCs after transplantation [10].

The development of tissue engineering technology has brought hope to address this issue. Research has shown that adult bone marrow has multi-potential mesenchymal stem cells (MSCs) that are easy to isolate and can be directly induced to differentiate into cardiac cells, fat cells, epithelial cells, endothelial cells, nerve cells, bone, cartilage, tendon, and bone marrow stromal cells under different conditions in vitro [11-13].

In this study, we tested whether MSCs have the potential to differentiate into corneal epithelial cells and whether they can be used to treat LSCD. We evaluated the characteristics of MSCs co-cultured with CSCs in a transwell system and further transplanted the induced MSCs seeded on an AM in an LSCD rat model to study the biologic functions of corneal surface reconstruction.

## Methods

### Rats

Male and female inbred Sprague Dawley (SD) rats aged 6 to 8 weeks were obtained from the Animal Facility of the Fourth Military Medical University, Xi’an, China. All the rats were treated according to the ARVO Statement for the Use of Animals in Ophthalmic and Vision Research.

### Antibodies and reagents

The following antibodies were obtained from Chemicon (Temecula, CA): CD29/34/45 mAbs. CK12/13 mAbs and phycoerythrin (PE) labeled CD34 were purchased from Santa Cruz (Santa Cruz, CA). Anti-CD44-fluoroscein isothiocyanate (FITC) was purchased from AbD Serotec (Raleigh, NC). Anti-CD45-FITC, anti-CD90-FITC, anti-CD34-PE, and anti-CD133-PE were purchased from Miltenyi Biotec (Auburn, CA). Anti-CD71-FITC and anti-CD29-PE came from BD PharMingen (San Diego, CA). FITC or PE labeled Mouse antirat IgG was purchased from BD Biosciences (Mountain View, CA). Normal Mouse antirat IgG was purchased from Sigma-Aldrich (St. Louis, MO). The transwell system (aperture 0. 45 μm) came from Millipore (Billerica, MA).

### Isolation and cultivation of mesenchymal stem cells

Sprague Dawley rats were sacrificed and the ends of the diverticula part of the femurs were cut and the bone marrow cavity was washed with phosphate buffered saline (PBS) containing heparin (2,500 U/ml). The fluid was slowly added to the 1.073 g/ml percoll separation and centrifuged (3,000 r, 30 min), and then the middle layer of mononuclear cells was collected. The collected cells were subsequently washed three times with PBS. The cells were resuspended in a DMEM/F12 medium containing 10% fetal bovine serum, 1% L-glutamine, 1% penicillin, and streptomycin and were seeded in a 75 ml bottle at a density of 2×10^6^/cm^2^, which was maintained in a humidified atmosphere of 5% CO_2_ at 37 °C [14]. The cells had half media changes every two to three days and were observed daily for cell proliferation and morphological characteristics under an inverted microscope.

### Cultivation of corneal stromal cells

The corneas of the rats were isolated from the eyeball along the limbal and were immersed in the digestive solution (0.25% pancreatin + 0.02% EDTA, 1:1) at 37 °C for 30 min. The epithelium was scraped and the endothelium was stripped separate from the cornea under microscope. Tissue explants without endothelium and the epithelium were cut into approximately 1 mm×1 mm and put into 15 ml centrifuge tubes with 2 mg /ml of collagenase added to digest for 1 h. The cells were cultured with a cultural medium of DMEM /F12 supplemented with 10% FBS, 1% L-glutamine, 1% penicillin, and streptomycin at a density of 1×10^6^/cm^2^. The culture condition was 37 °C and 5% CO_2_, and the culture medium was changed every two to three days [15].

### Induction of mesenchymal stem cells by corneal stromal cells

Second or third generation rat MSCs and CSCs were co-cultured within a transwell system according to Millipore's note (aperture 0.45 μm) with MSCs on the lower layer. The system was placed in a 5% CO_2_, 37 °C incubator to culture for seven days. Cells had half media changes every other day and were observed under an inverted microscope daily.

### Flow cytometric analysis

The third generation of MSCs were harvested and incubated with the mAb for 30 min at 4 °C in the dark and then washed twice in a flow cytometry buffer immediately before cytometry data acquisition. FITC or PE labeled mouse antirat IgG was used as the negative control. Samples were acquired using flow cytometry and analyzed with the accompanying software (CellQuest). Ten thousand cells were analyzed in each sample.

### Immunofluorescence staining

Rat monoclonal CD29, CD34, CD45, and CK12 were used to identify MSCs by immunofluorescence staining. MSCs were fixed in 4% paraformaldehyde and then rinsed with PBS three times for 5 min each and with 0.5% Triton-X100-PBS three times, and were then incubated with 2% BSA in PBS for 30 min. Before incubation in a moist chamber overnight at 4 °C, primary antibodies were applied to the samples. The samples were then rinsed three times for 5 min each in 0.5% Triton-X100-PBS. Finally, samples were mounted in a glycerol carbonate buffer for detection and photography. Negative controls were incubated with mouse antirat IgG instead of the primary antibody.

### Induced mesenchymal stem cells seeded on amniotic membrane

Human placentas were harvested at the time of cesarean section after obtaining informed consent from donors who had negative serologic tests for hepatitis B and C virus, syphilis, and human immunodeficiency virus, and the amnion was handled as previously reported [16]. Under sterile conditions, the amnion was separated from the chorion by blunt dissection and washed with PBS containing penicillin 1000 U/ml, streptomycin 20 mg/ml, and amphotericin B 2.5 mg/ml. Under a laminar air hood, the amnion was flattened onto nitrocellulose paper, with the epithelium scraped. The paper with the adherent membrane was then cut into pieces of 4×4 cm^2^ and stored at −80 °C in a sterile vial containing glycerol and modified DMEM.

To culture MSCs, the AM was fixed on a scaffold, which was made using iron wire in a Petri dish to prevent the AM from floating. A small amount of DMEM/F12 medium was added to keep the AM wet. MSCs in the transwell system that had been cultured for seven days were digested and inoculated on the AM surface at a density of 1×10^6^/cm^2^ and cultured in the 5% CO_2_, 37 °C incubator, without adding more culture medium, for the initial 4 h. After 4 h, DMEM containing 10% FBS was added to submerge the AM, and the MSCs on AM were then cultivated at the air-liquid interface for seven days to form an epithelial graft. The culture media was very gently changed every two days.

### Animal model of limbal stem cell deficiency 

LSCD was generated in the right eye of each rat (48 rats). The rats received intraperitoneal anesthesia with 1% pentobarbital sodium (40–50 mg/kg), and ocular anesthesia with tetracaine complemented with topical proparacaine. A filter paper ring with a diameter of 7 mm was saturated with 0.5 mol/l NaOH and was placed on the corneal limbus for 20 s, followed by rinsing with 0.9% saline for 1 min. Antibiotic drops were applied to the injured eyes three times a day. Observation of changes in the anterior segment was performed periodically with a slit-lamp.

### Corneal surface evaluation and grouping

Each injured cornea was examined daily using a slip-lamp microscope. Sodium fluorescein solution was used to indicate the extent of the corneal epithelial defects. The corneal surface was examined for opacity, neovascularization, and fluorescence staining by slit lamp examination once a week. The criterion for ocular surface evaluation was the same as described previously (Table 1) [17]. After alkali injury, the rats with corneal opacity grades ≥ 2, cornea neovascularization grades ≥ 2, and fluorescence staining grades ≥ 3 were included in the study.

**Table 1:** Rat’s corneal score criterion after alkali burn.

**Scores**	**0**	**1**	**2**	**3**	**4**
Corneal opacity	transparent	slight opacity with visible iris texture	moderate opacity with unclear iris texture	severe opacity pupil can be seen vaguely	very severe opacity pupil can not be seen
Neovascularization grades	No	2 mm within limbal	around cornea≤1/2 quadrant	around cornea>1/2 quadrant	whole cornea
Fluorescence staining area	No	≤1/4 quadrant	1/4<area≤1/2quadrant	1/2<area≤3/4quadrant	>3/4quadrant

The selected 48 rats were randomly divided into four groups: the control group (Group 1, n=12), simple AM group (Group 2, n=12), uninduced MSCs seeded on the AM group (Group 3, n=12), and MSCs induced by CSCs seeded on the AM group (Group 4, n=12).

### Surgical procedure for transplantation

The damaged corneal epithelium was carefully keratectomized under anesthesia and then rinsed with normal saline. The grafts were transplanted onto the corneal surface using 10–0 nylon sutures, with the seeded cells face up. After transplantation, a 0.05% dexamethasone gentamicin solution was applied. Finally, the lids were sutured and the sutures were removed seven days later. Dexamethasone gentamicin was applied to the right eye of each rat three times a day. The ocular surface of each rat was evaluated for ten weeks after surgery. The rats were sacrificed and the corneas were extracted and processed for H&E and immunofluorescence staining.

### Statistical analysis

All data obtained from corneal surface evaluation were processed by an SAS software package and analyzed by an LSD-*t*-test; p<0.05 was regarded as statistically significant.

## Results

### Mesenchymal stem cells culture

**Figure 1:**
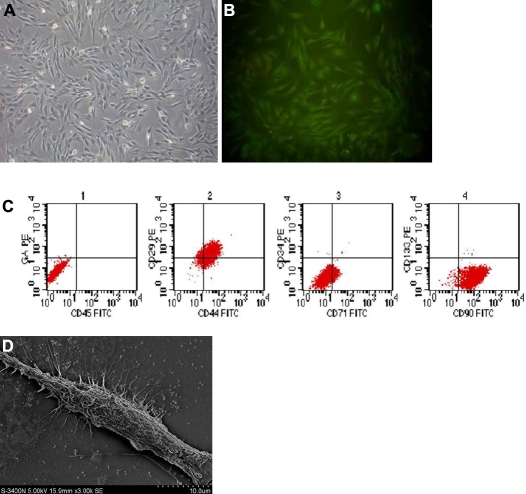
Subcultured mesenchymal stem cells (MSCs) in vitro. **A**: Phase contrast micrographs of mesenchymal stem cells cultured on day seven (40×). **B**: Immunofluorescence staining of CD29 was positive. **C**: Flow cytometric analysis showed that the positive rates of CD29, CD34, CD44, CD45, CD71, CD90, and CD133 were 81.56%, 0.10%, 88.77%, 0.17%, 10.02%, 98.43%, and 1.56%, respectively. **D**: Scanning electron microscopy (SEM) of MSCs showed a long spindle-shaped appearance with rich processes (1000×).

Three days after culture, the bone marrow cells began to stretch. The non-adherent cells were removed by changing the cultured media. The spindle-like MSCs distributed widely. There were larger clones on day seven, and the cells grew rapidly and became fibroblast-like with rich cytoplasm and a large nucleus. The cells were tightly adherent and not easy to digest. After three subcultures, the cells were confluent and had a spiral whorl-like outlook (Figure 1A).

### Mesenchymal stem cells identification

Immunofluorescence staining showed that MSCs expressed the mesenchymal cell marker CD29 (Figure 1B), but did not express hematopoietic stem cell markers CD34 or CD45. Keratin 12, which is the corneal epithelial-specific expression, signs were negative. Flow cytometry analysis showed that the positive incidence of CD29, CD44, and CD90 were 81.56%, 88.77%, and 98.43%, respectively, while the positive incidence of CD34, CD45, CD71, and CD133 were 0.10%, 0.17%, 10.02%, and 1.56%, respectively (Figure 1C). Under a scanning electron microscope, MSCs were elongated and spindle-like with rich cell processes (Figure 1D).

### Corneal stromal cell culture

After culturing for two days, CSCs extended and showed a spindle or irregular triangles outlook. An oval-shaped nucleus was in the middle of the cell and the cytoplasm extended outward. Three days later, the number of cells gradually increased and the cells grew rapidly. The primary cells became confluent on day seven. After passage, the second generation of CSCs was used for the co-culture system.

### Induced mesenchymal stem cells by corneal stromal cells in a transwell system

**Figure 2:**
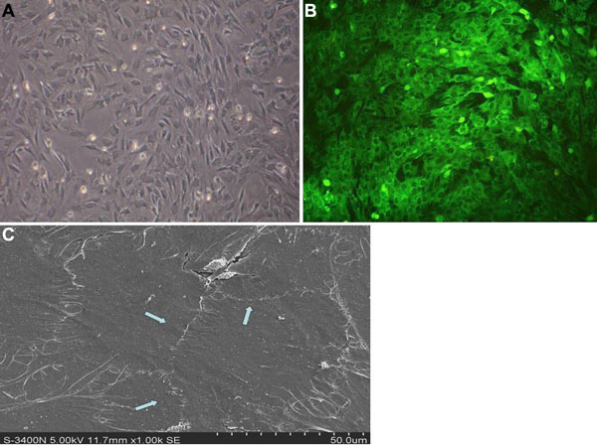
Induced MSCs by corneal stromal cells. **A**: Phase contrast micrographs of induced MSCs cultured on day seven (40×). **B**: Expression of CK12 on induced MSCs was positive. **C**: SEM of induced MSCs showed there were large numbers of epithelial-like cells that were connected even in a patch and the structure of the cellular tight junction was clear (arrow; 1000×).

MSCs and CSCs were cocultured in a transwell system for seven days. About half of the induced MSCs’ morphology was square and flat and they gradually became larger (Figure 2A). The number of long spindle cells was reduced. Immunofluorescence staining showed that the expression of keratin CK12 on the surface of many cells was positive (Figure 2B); SEM showed that there were a large number of epithelial-like cells, which were connected, even in a patch, and the structure of the cellular tight junction was clear (Figure 2C). Morphological analysis showed that this tight junction was a characteristic structure of the epithelial cells, and suggested that some MSCs had been induced to transdifferentiate into corneal epithelial cells in a transwell system.

### Epithelial cells graft

**Figure 3:**
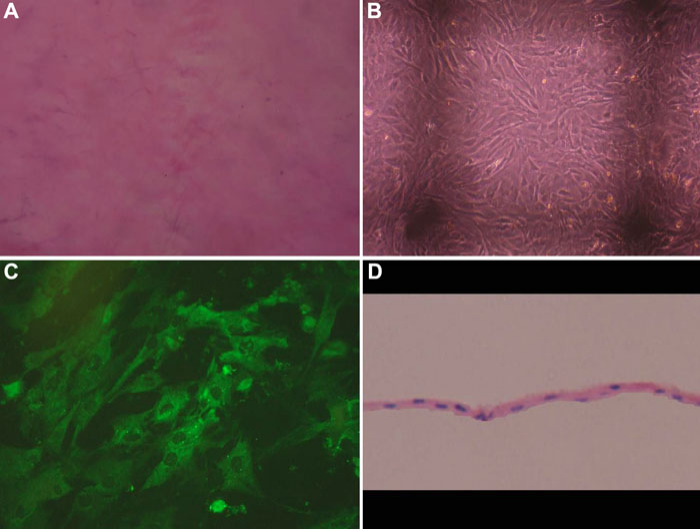
Induced MSCs on the amniotic membrane (AM). **A**: Phase contrast micrographs of the AM after trypsin digestion showed no cells left. **B**: Morphology of MSCs seeded on the AM for three days were clear and did not significantly change compared with the pre-vaccination (40×). **C**: Immunofluorescence staining showed the epithelial-like cells on the AM cultured for three days expressed CK12. **D**: Section of epithelial cell graft by hematoxylin and eosin (H&E) staining showed epithelial like cells on the collagen fibers of AM.

There were no cells left on the AM after trypsin digestion (Figure 3A). The induced MSCs were inoculated on the AM at a density of 1×10^6^/cm^2^. The cells began to adhere after 4 h, and fully extended after 28–48 h. The cells grew rapidly and had good viability under the phase contrast microscope. The cells formed single-layer integration on day three, which were clear and did not significantly change morphology compared with the pre-vaccination (Figure 3B); immunofluorescence staining showed the epithelial-like cells on the AM expressed CK12 (Figure 3C). It began to form a stratified epithelium-like structure on days five through seven, and the cell’s structure could not be discerned easily. The cell growth was so dense on day ten that the structure was unclear and the older cells increased. Therefore, the induced MSCs seeded on the AM on day seven were transplanted. HE staining showed that there were epithelial-like cells on the collagen fibers of the AM, and the organizational structure was compact (Figure 3D).

### Surgical effects in the rat alkali burn model

Two weeks after alkali injury, all injured eyes showed moderate to severe corneal opacity with neovascularization and epithelial defects. Partial conjunctivalization was found in the corneal epithelium of some of the rats. Rats with corneal opacity ≥ 2 (mean 2.42±0.5), cornea neovascularization ≥ 2 (mean 2.42±0.5), and fluorescence staining ≥ 3 (mean 3.25±0.44) were selected for the study. Four rats were not included in the experiment because of a corneal perforation in two and a mild corneal alkali burn in the other two.

**Figure 4:**
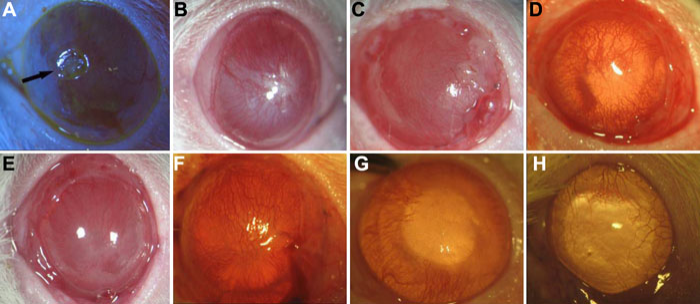
Surgical effects of different treatments in the rat corneal alkali burn model. **A**, **B**: Control group (**A**). Four weeks after transplantation, corneal epithelial defect size > 1/2 quadrant and neovascularization grew rapidly and involved 1/2 quadrants, with ulcer emerged (arrow) (**B**). The new blood vessels grew into the whole cornea with extremely severe opacity ten weeks after transplantation. **C**, **D**: AM group (**C**). Moderate to severe corneal opacity four weeks after transplantation (**D**). Corneal neovascularization grew into nearly 3/4 quadrants ten weeks after transplantation. **E**, **F**: Non-induced MSC group (**E**). Moderate corneal opacity with local stromal scar was seen four weeks after surgery (**F**). Conjunctivalization with apparent neovascularization was found in 1/2–2/3 quadrant ten weeks after surgery. **G**, **H**: Induced MSC group (**G**). The corneal opacity was mild and there were no signs of conjunctival epithelium growing into the cornea four weeks after transplantation (**H**). Cornea kept transparent and the neovascularization was within 2 mm from limbal ten weeks after surgery.

**Figure 5:**
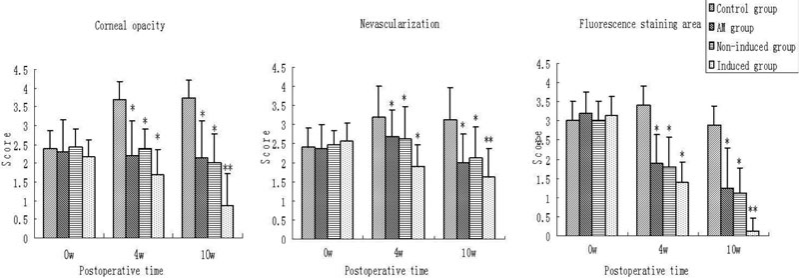
Corneal scores after different treatments of the corneal alkali burn injury of the rats ($\bar{X}$±s). *p<0.05 versus control, **p<0.01 versus control

Four weeks after transplantation, 80% of the rats in the control group showed extremely severe corneal opacity (3.70±0.48) and obvious neovascularization (3.22±0.79; Figure 4A). The corneal epithelium was completely defected in the rats with positive fluorescein staining and two had corneal ulcers (3.41±0.52). However, the AM was dissolved 2–3 weeks after transplantation in the other three groups. Four weeks after surgery, more severe corneal opacity was seen in Group 2 (Figure 4C) and Group 3 (Figure 4E; 2.20±0.92, 2.40±0.52, respectively) than in Group 4 (Figure 4G; 1.71±0.68). Neovascularization invasion was 2.73±0.68 and 2.63±0.84 in Group 2 (Figure 4C) and Group 3 (Figure 4E), respectively, which were both higher than Group 4 (Figure 4G; 1.92±0.57). Fluorescence staining showed that the scores in Groups 2, 3, and 4 were all statistically lower than the control group (Figure 4 and 5; Table 2).

**Table 2:** Corneal scores after different treatments for rat’s corneal alkali burn injury ($\bar{X}$±s).

**Time**	**Group**	**Corneal opacity**	**Neovascularization grades**	**Fluorescence staining area**
Pre-operative	G1 (n=12)	2.38±0.49	2.41±0.50	3.00±0.42
	G2 (n=12)	2.29±0.87	2.37±0.63	3.20±0.56
	G3 (n=12)	2.16±0.46	2.56±0.48	3.15±0.47
	G4 (n=12)	2.41±0.51	2.46±0.39	3.00±0.51
Postoperative (4wk)	G1 (n=10)	3.70±0.48	3.22±0.79	3.41±0.52
	G2 (n=10)	2.20±0.92*	2.73±0.68*	1.92±0.74*
	G3 (n=10)	2.40±0.52*	2.63±0.84*	1.81±0.79*
	G4 (n=10)	1.71±0.68*	1.92±0.57*	1.41±0.52*
Postoperative (10wk)	G1 (n=8)	3.75±0.46	3.13±0.84	2.88±0.99
	G2 (n=8)	2.12±0.99*	2.00±0.76*	1.25±1.04*
	G3 (n=8)	2.01±0.76*	2.12±0.83*	1.12±0.64*
	G4 (n=8)	0.88±0.84** #	0.75±0.71** #	0.13±0.35** #

G1: control group; G2: AM group; G3: Non-induced MSC group; G4: Induced MSC group. *p<0.05 versus control, **p<0.05 versus Group2 and 3, ^#^p<0.05 versus 4 weeks.

Ten weeks after transplantation, the pupils of eight rats could not be seen and five appeared to be scarred (3.75±0.46) in Group 1 (control). Neovascularization was seriously invasive (3.13±0.84), while fluorescein staining declined slightly compared with week four (Figure 4B; 2.88±0.99). In Group 2, the opacity of the cornea was moderate in six rats and severe in two rats (2.12±0.99). Neovascularization was growing slower than the control group (2.00±0.76). However, partial conjunctivalization was found in the corneal epithelium of all rats and the corneal epithelial defected areas were less than 1/4 quadrants in eight rats (Figure 4D; 1.25±1.04). The results of Group 3 were similar to Group 2 (Figure 4F; Table 2); the scores of the three parameters were 2.12±0.84, 2.0±0.76, and 1.13±0.64, respectively. In Group 4, the corneal epitheliums in 75% of the rats were transparent, and neovascularization was limited within 2 mm of the limbus. The fluorescence staining was negative, which indicated that the cornea had been reconstructed by reepithelization (Figure 4H). The scores of the three parameters were 0.88±0.84, 0.75±0.71, and 0.13±0.35, respectively (Figure 4 and Figure 5; Table 2).

LSD-t analysis showed that there were significant differences between Group 1 and the three other groups on corneal opacity, neovascularization grades, and fluorescence staining at both four and ten weeks after transplantation (p<0.05). Meanwhile, the differences were significant between Group 4 and Groups 2 and 3 at ten weeks (p<0.05), but were not significant between Groups 2 and 3 (p>0.05; Figure 5; Table 2). The mean differences of the three parameters of Group 4 were significant between week four and week ten (p<0.05; Table 2).

### Hematoxylin and eosin staining

**Figure 6:**
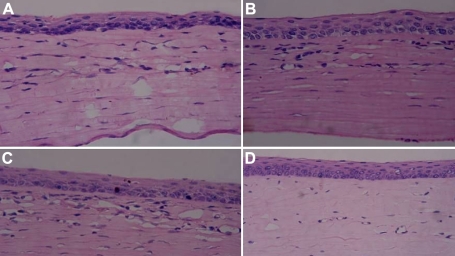
H&E staining of four groups ten weeks after transplantation. **A**: Control group: Epithelial structure was disordered, and numerous inflammatory cells and neovascularization emerged. **B**: AM group: Epithelium was integrated and some inflammatory cells infiltrated in the stroma. **C**: Non-induced MSC group: Incomplete epithelial hyperkeratosis and inflammatory cell infiltration. **D**: Induced MSC group: Epithelium was intact with a few lymphocytes and neovascularization infiltration. The magnification was 200×.

Ten weeks after surgery, in Group 1, the cornea was severely damaged without intact configuration with numerous inflammatory cells and neovascularization infiltration in the stroma (Figure 6A). In Groups 2 and 3, the cornea exhibited epithelial and stromal defects in various degrees. The epithelium was integrated and the stromal layer had many inflammatory cells infiltration (Figure 6B,C). In Group 4, the epithelium was intact with a few lymphocytes and neovascularization infiltration (Figure 6D).

### Immunohistochemistry staining

**Figure 7:**
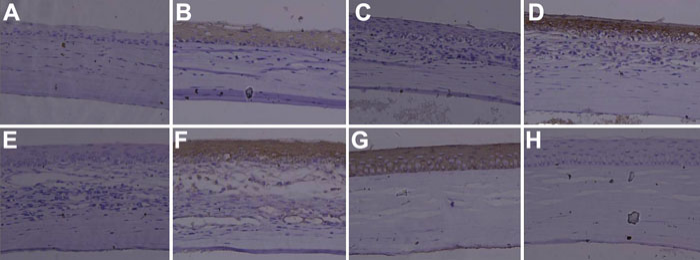
Immunohistochemistry staining of the four groups ten weeks after transplantation. **A**, **C**, and **E**: The expression of CK12 was negative in the control group, AM group, and non-induced MSC group; **G**: The expression of CK12 was positive in the induced MSC group; **B**, **D**, and **F**: The expression of CK13 was positive in the control group, AM group, and non-induced MSC group; **H**: The expression of CK13 was negative in the induced MSC group, indicating that the epithelium of the induced group were the corneal source within the ten week observation period. The magnification was 200×.

Ten weeks after surgery, the corneal epitheliums of the four groups were evaluated by immunofluorescence staining of CK12 and CK13. In the induced MSCs group, CK12 staining was positive, while CK13 was negative, suggesting that the epithelium was corneal but not conjunctival-derived. In the three other groups, CK12 was negative, while CK13 was positive, suggesting that the epithelium may be conjunctival-derived (Figure 7).

### Confocal laser corneal microscopy

**Figure 8:**
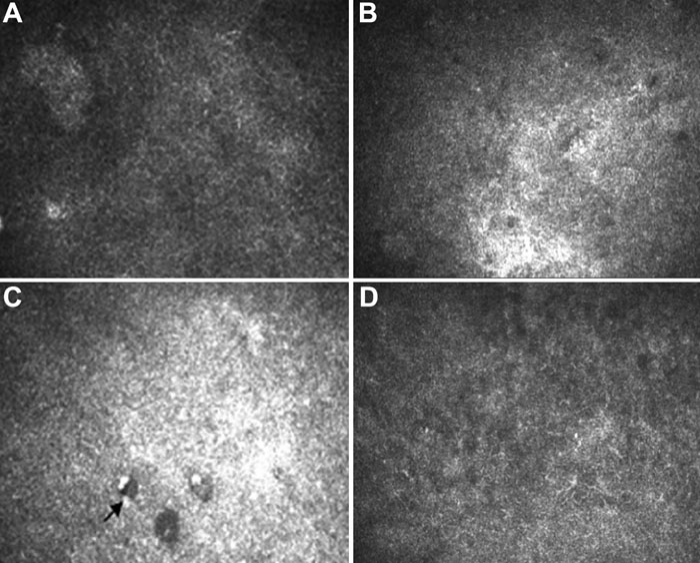
Confocal laser corneal microscopy images of the rats' corneal epithelium ten weeks after transplantation. **A**: Epithelium cells were well arranged and integrated closely in the normal cornea. **B**: The structure of epithelial cells was not clear with ambiguous boundary in the control group. **C**: The epithelial cells of the AM group were disarranged with an enhanced reflection and cavities (arrow). **D**: The epithelial cells of the induced MSC group were integrated with clear boundaries.

**Figure 9:**
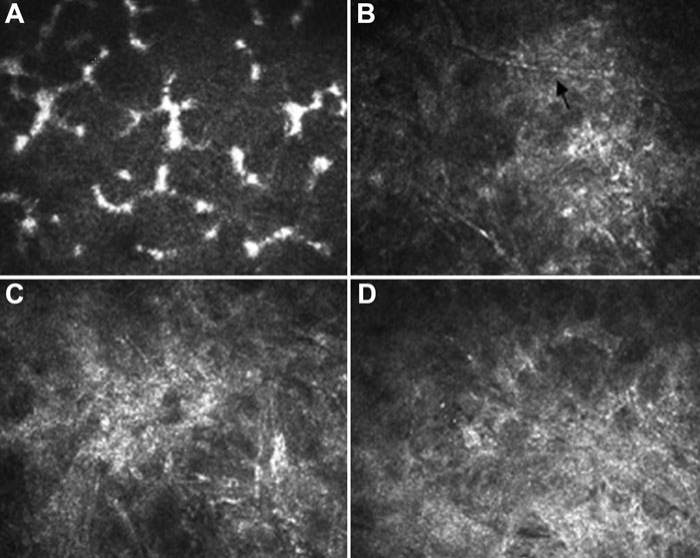
Confocal laser corneal microscopy images of the rats' corneal stroma ten weeks after transplantation. **A**: Normal corneal stromal cells were well arranged. **B**: Neovascularization (arrow) can be seen in the anterior matrix with the proliferation of stromal cells in the control group. **C**: Stromal cells proliferated and inflammatory cells existed in the anterior stroma of the AM group. **D**: Stromal cells also proliferated while a few neovascularizations appeared in the stroma of the induced MSC group.

Ten weeks after transplantation, in the control group, the cornea cells’ structure was disordered with an ambiguous boundary. Neovascularization appeared in the anterior matrix, while the posterior matrix remained normal. The epithelial cells of the AM group were disarranged with an enhanced reflection, neovascularization and cavity existed in the anterior stroma. In the induced MSCs group, the surface epithelial cells were integrated closely with clear boundaries. There was some neovascularization in the stroma, though the stroma cells proliferated moderately (Figure 8 and Figure 9).

## Discussion

The stability of the corneal surface plays an important role in maintaining the transparency of the cornea. Above all, LSCs are the source of corneal epithelial cell regeneration. The deficiency of LSCs will lead to serious ocular surface abnormalities, resulting in corneal opacity, diminished vision, etc. At present, there is still no satisfactory treatment for these diseases. It is reported that a limbal stem cell autograft has some positive effects [1,2]. However, if both eyes have serious surface damage, the source of healthy LSCs will be lost. In recent years, studies have shown that the MSCs in adult bone marrow have multilineage differentiation potential [11-13,18]. It can be induced to differentiate into various cells under different conditions in vitro [14,19,20]. These cells have many advantages, such as adequate sources, convenient material, and multilineage differentiation potential, which can effectively avoid the ethical issues that embryonic stem cells may face. And because they are self-organization without rejection, they may have better prospects in clinic use. MSCs have both endothelial and epithelial tissue coding genes [12] and can be induced to differentiate into epithelial or epithelial-like cells in the micro-environment both in vitro and in vivo [21-24].

The cornea matrix is the micro-environment of the corneal epithelium and LSCs, and plays an important role in maintaining the characteristics of corneal epithelial cells. CSCs can secrete some kinds of autocrine and paracrine cytokines, which would maintain the normal differentiation and proliferation of LSCs with no access to the ultimate division. Recently, it was reported that the niche structure of CSCs has the ability to induce non-corneal epithelial cells to differentiate into corneal epithelial cells [25]. In a transwell co-culture system, which can imitate the cell growth environment in vivo to some extent, two kinds of cells will not contact directly, and the lipid membrane of 0.45 aperture can guarantee that cytokines will pass it while the normal cells will not; therefore, there will be no cell contamination. Cytokines produced by stroma cells formed a micro-environment, which promotes MSCs to differentiate into the corneal epithelium. After culturing for one week with CSCs, the induced MSCs can express specific keratin CK12, which are generally believed to be the specific marker for mature cornea epithelial cells [26]. SEM also proved that induced MSCs had an epithelial-cell look, and that the structure of cellular tight junction were clear, suggesting that some MSCs had been induced to differentiate into corneal epithelial cells. However, not all the MSCs had been induced to epithelial-like cells. In the experiment, we observed that about half of the cells had morphological changes after co-culture. The exact efficiency of the transdifferentiation and the factors that influence the results of transdifferentiation require further study.

Our study aims to find a better and more convenient seed cell for corneal tissue engineering. We used MSCs to replace LSCs, and found that they could be induced to corneal epithelial cells in vitro by CSCs, in accordance with the other article [27]. Next, we constructed a corneal epithelial graft using an AM as the growth platform of the cells. The AM has translucent organization, which can provide the constitutive basement membrane, and contains the same plate of body as the cornea and VII collagen, laminin, fibronectin protein, and a variety of integration [28]. Laminin can provide a good micro-environment for cells and plays an important role in epithelial paste, cell adhesion, and the maintenance of the stem cell activity of nerve growth factor NGF [29-31]. Tsai planted healthy limbus epithelial tissue on the AM, and transplanted it back to the eye two to three weeks after culture. After follow-up for 15 months, the vision acuity of the five patients (six cases) was markedly improved [32]. Koizumi et al. [33,34] proved that the use of non-epithelial AM and 3T3 as a feed cell to culture LSCs leads to a better effect after transplantation. These studies are mostly on allotransplantation; thus, graft rejection will influence the effect of treatment, becoming a major challenge. Our results suggested that the AM can be a carrier of induced MSCs. As a platform for cell growth, the AM does not change the morphology and characteristics of the cell itself. Induced MSCs inoculated on an AM still expressed CK12, which was proven by immunofluorescence staining.

Our results showed that compared with other groups, the induced MSCs group can significantly improve corneal opacity and neovascularization invasion. Ten weeks after the transplantation of the induced MSCs, the corneal epithelium was transparent in 75% of the rats, neovascularization was limited within 2 mm of the limbus, and fluorescence staining and inflammatory cells infiltration were negative. While in the non-induced MSCs and AM transplantation group, the corneal epithelium appeared partially conjunctivalized and various degrees of inflammatory response could be seen. However, the corneal opacity and epithelial defected area were gentler in the Groups 2 and 3 than in the control group. Results of HE staining showed that the corneas in Groups 1, 2, and 3 had epithelial coverage, but this was more likely conjunctival epithelial derived rather than corneal epithelium derived, which was proven by immunohistochemistry results. Corneal confocal laser microscopy results were in line with the above-mentioned results. The results here showed that cultured corneal epithelial cells autologous transplantation seems to be an ideal way to treat corneal surface diseases, which could prevent both allogeneic transplant rejection and ethical problems, though the long-term prognosis needs further study.

In conclusion, our findings clearly indicate that MSCs can be cultivated and induced to transdifferentiate into corneal epithelial cells by CSCs in a transwell system. MSCs seeded on a human AM can form an epithelial graft that can be transplanted and that reconstruct the corneal epithelial surface of a rat’s eyes with an alkali burn injury. However, there are still several problems left, such as whether the extrinsic transplanted MSCs can keep the epithelial cell characteristics in the recipient eye and whether these cells have stem cell function to maintain the normal corneal homeostasis for long-term observation. Further studies are needed to provide more reliable proof for the clinical application of MSCs in the future.
